# Well Plate–Based Localized Electroporation Workflow for Rapid Optimization of Intracellular Delivery

**DOI:** 10.21769/BioProtoc.5037

**Published:** 2024-07-20

**Authors:** Cesar A. Patino, Sevketcan Sarikaya, Prithvijit Mukherjee, Nibir Pathak, Horacio D. Espinosa

**Affiliations:** 1 Department of Mechanical Engineering, Northwestern University, Evanston, IL, USA; 2 Theoretical and Applied Mechanics Program, Northwestern University, Evanston, IL, USA

**Keywords:** Electroporation, Intracellular delivery, Deep learning, Multiplexing, Cell engineering, Transfection

## Abstract

Efficient and nontoxic delivery of foreign cargo into cells is a critical step in many biological studies and cell engineering workflows with applications in areas such as biomanufacturing and cell-based therapeutics. However, effective molecular delivery into cells involves optimizing several experimental parameters. In the case of electroporation-based intracellular delivery, there is a need to optimize parameters like pulse voltage, duration, buffer type, and cargo concentration for each unique application. Here, we present the protocol for fabricating and utilizing a high-throughput multi-well localized electroporation device (LEPD) assisted by deep learning–based image analysis to enable rapid optimization of experimental parameters for efficient and nontoxic molecular delivery into cells. The LEPD and the optimization workflow presented herein are relevant to both adherent and suspended cell types and different molecular cargo (DNA, RNA, and proteins). The workflow enables multiplexed combinatorial experiments and can be adapted to cell engineering applications requiring in vitro delivery.

Key features

• A high-throughput multi-well localized electroporation device (LEPD) that can be optimized for both adherent and suspended cell types.

• Allows for multiplexed experiments combined with tailored pulse voltage, duration, buffer type, and cargo concentration.

• Compatible with various molecular cargoes, including DNA, RNA, and proteins, enhancing its versatility for cell engineering applications.

• Integration with deep learning–based image analysis enables rapid optimization of experimental parameters.

## Background

Intracellular delivery of molecular cargoes allows for a wide variety of cell manipulation tasks with applications ranging from cell engineering and gene editing to cell therapy [1,2]. Given the wide implications of intracellular delivery for fundamental biology research and therapeutics, several technologies have emerged to achieve this task with high efficiency and precision that can be classified into two broad categories: i) direct delivery and ii) carrier-mediated delivery [3]. In direct-delivery methods, the cell membrane is permeated by subjecting it to physical disturbances such as mechanical forces [4–7], electric fields [8–10], or thermal shock [11]. Conversely, carrier-mediated delivery methods rely on chemical interactions between the cell membrane and the nanocarrier, which trigger endocytosis and encapsulation of the molecular cargo [3]. Examples of nanocarriers include lipid vesicles [12], spherical nucleic acids [13], and polymer nanoparticles [14]. Direct delivery methods offer more control and tunability over the membrane permeation process, which can result in tighter dosage precision compared with carrier-mediated methods [1,15].

Electroporation is one of the most widely used direct-delivery methods because of its ease of use and high throughput. In bulk electroporation systems, cells are placed in a cuvette with embedded electrodes that subject a suspension of cells to an electric field that induces the formation of pores across the cells when the electric potential across the membrane [transmembrane potential (TMP)] reaches a certain threshold. However, high applied voltages are required to achieve threshold TMP in bulk electroporation systems, which results in high cell mortality. Furthermore, the variability in the spatial distribution of the cells in the cuvette results in heterogeneous electric field conditions from cell to cell, which reduces dosage precision [1]. To circumvent these limitations, researchers developed electroporation systems that confine the electric field to a small fraction area of the cell membrane by interfacing cells with nanoscale orifices prior to the application of electric pulses [9,16,17]. The resulting highly localized electric fields enable the application of much lower voltages compared to bulk systems to achieve threshold TMPs, which results in higher viability. Examples of nanoscale features used in localized electroporation systems include nanochannel membranes [18,19], nanopipettes [20], nanostraws [21], and nanofountain probes [22].

Localized electroporation systems have been shown to be efficient in delivering various payloads (e.g., nucleic acids, plasmids, proteins, and nanoparticles) as well as sampling intracellular contents from cells [23,24]. The performance of localized electroporation systems depends on the electric field conditions, the mechanical properties of the cell membrane, the ionic environment in the surrounding fluid, and the physical properties of the molecular cargo (i.e., size and charge) [2,3]. While these systems offer significant advantages, they also pose challenges in scalability in the number of cells and require high cargo concentrations, which may limit their applicability in certain scenarios. Consequently, several parameters must be optimized to achieve the desired electroporation performance, including both delivery efficiency and cell health. To expedite the optimization process, we designed a well plate–based localized electroporation device (LEPD) equipped with multiplexing capabilities [9]. The multi-well LEPD system allows for tunability in the electric pulse conditions across the rows of the device and the molecular cargo or buffer conditions across columns. Furthermore, we integrated the LEPD with automated imaging and deep-learning enhanced segmentation to analyze the morphology of cells following delivery in addition to delivery efficiency and viability. The integrated approach provides a flexible platform suitable for adherent or suspended cell manipulation and analysis. In this protocol, we detail the experimental workflow of the LEPD spanning from device fabrication to optimization of plasmid delivery.

## Materials and reagents

1. Well-plate electrode assembly
  - Custom printed circuit board (PCB) with gold electrode pads (Infinitesimal LLC, infinitesimal.llc.hde@gmail.com)
  - Custom PCB with through-holes (Infinitesimal LLC, infinitesimal.llc.hde@gmail.com)
  - Push-fit PCB receptacle (Mill-Max, catalog number: 0350-0-15-15-07-27-10-0)
  - Au-coated pins (straight pin or nail-head pin) (Mill-Max, catalog number: 3580-1-00-15-00-00-03-0)
  - Bottomless well plate (Greiner Bio-One, catalog number: 662000-06)
  - Biopsy punch, 12 mm (Acuderm, catalog number: P1250)
  - Double-sided pressure adhesive tape (Adhesives Research, catalog number: MH-90880)
  - Cutting board
  - Razor blade
  - Marker
2. Device assembly
  - Track-etched polycarbonate (PC) membranes (Itpi4, catalog number: 1000M25/620N401/7525)
  - Cloning cylinders (Millipore Sigma, catalog number: CLS31668)
  - Double-sided pressure adhesive tape (Adhesives Research, catalog number: MH-90880)
  - Biopsy punch, 6 mm (Miltex, catalog number: 33-36)
  - Acetone (Sigma-Aldrich, catalog number: 179124)
  - 70% EtOH/H_2_O
  - Nitrogen (N_2_) (Airgas, catalog number: UN1066)
  - Oxygen (O_2_) (Airgas, catalog number: UN1072)
  - Scissors
3. Electroporation: low conductivity electroporation (EP) buffer (Eppendorf, catalog number: EW-36205-62)
4. Surface treatment and cell culture
  - Fibronectin from human plasma (0.1% solution) (Sigma-Aldrich, catalog number: F0895)
  - Dulbecco’s modified Eagle medium (DMEM), high glucose, pyruvate (Gibco, catalog number: 11995065)
  - Fetal bovine serum (FBS) (Gibco, catalog number: A3160501)
  - Penicillin-Streptomycin (Pen-Strep) (10,000 U/mL) (Gibco, catalog number: 15140148)
  - Trypsin-EDTA (0.25%), phenol red (Gibco, catalog number: 25200056)
  - Nunc^TM^ cell culture–treated multi-dishes, 6-well plates (Thermo Fisher Scientific, catalog number: 140675)
  - Phosphate buffered saline (PBS) (Gibco, catalog number: 10010023)
  - HeLa cells (ATCC, catalog number: CCL-2)
5. Plasmid: eGFP plasmids (Addgene, stock concentration 1,000 ng/μL)
6. Automated imaging: Hoechst 33342 nuclear stain (Invitrogen, catalog number: H3570)

## Equipment

1. Electronics box for pulse generation and resistance measurements along with pulse control software (Infinitesimal LLC, infinitesimal.llc.hde@gmail.com)
2. Inverted microscope (Nikon Ti-E) equipped with 4×, 10×, and 20× objectives, fluorescent light source, an epi-filter cube with a DAPI filter, XYZ motorized stage, and a CMOS camera (Andor Zyla) for image acquisition
3. Computer for setting pulse parameters and image processing
4. Laboratory centrifuge (Thermo Fisher Scientific, catalog number: 75007200)
5. Sonicator (Ultrasonic Cleaning Bath, Branson, catalog number: 3800)
6. Oxygen plasma cleaner (Harrick Plasma, catalog number: PDC-32G)

## Procedure

- **Well-plate electrode assembly**
  1. Place the PCB on the double-sided adhesive tape laid on a flat surface and use a razor blade to carefully cut the tape around the PCB’s edges, ensuring it matches the PCB’s dimensions.
  2. Place the top PCB on top of the tape and use it as a stencil to mark the location of the wells using a marker (Figure 1B).
  3. Use a plotter cutter or a biopsy punch (10–15 mm) to cut holes at the markings from the previous step (Figure 1C).
  4. Remove one side of the adhesive tape by carefully inserting a razor blade at a corner and pulling the film with clean tweezers. Place the bottom PCB adjacent to the tape facing up (Figure 1D).
  5. Use two tweezers to hold the tape from both sides, carefully align the tape to the bottom PCB by ensuring the holes on the tape are concentric to the Au pads on the PCB, and place the tape on the surface of the PCB.
  6. Press down firmly on the adhesive tape to ensure a tight seal by using a rigid cylinder to roll over the adhesive film while applying force onto the surface (Figure 1E).
  7. Remove the remaining protective film from the double-sided adhesive tape by separating the film from the adhesive tape using a razor blade and removing the laminate with a tweezer (Figure 1F).
  8. Place the bottomless well plate on the surface while ensuring proper alignment of the wells to the Au pads. Firmly press down on the well plate to ensure a tight seal (Figure 1G).
  9. Insert the receptacles onto the holes of the top PCB firmly and insert the Au-coated pins into the receptacles (Figure 1H and I).
  10. Carefully position the pin headers at designated connection points on the top PCB. Solder PCB pin headers to the top PCB to connect the assembled well-plate electrodes to the function generator.
  11. The aforementioned steps of well plate assembly are demonstrated in Video 1.
    **Figure 1.**
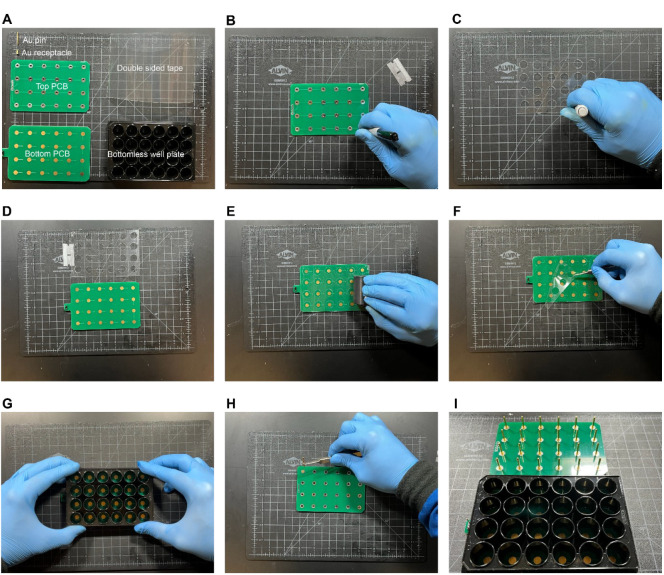
Assembly of well plate electrodes. (A) Materials required for the assembly procedure include an Au pin and receptacle, top and bottom printed circuit board (PCBs), double-sided adhesive tape, and a bottomless well plate. (B) Top PCB used as a stencil to mark the locations to cut holes (C) using a biopsy punch. (D) Tape after removal of the protective film is aligned with the bottom PCB. (E) A roller is used to compress the tape and the bottom PCB to form a tight seal. (F) The remaining protective film is removed from the adhesive tape. (G) The bottomless well plate is aligned to the bottom PCB pads and pressed firmly to ensure a tight seal. (H) Receptacles and pins are inserted into the through-holes of the top PCB. (I) Image of the fully assembled well-plate electrodes.
    **Video 1.**
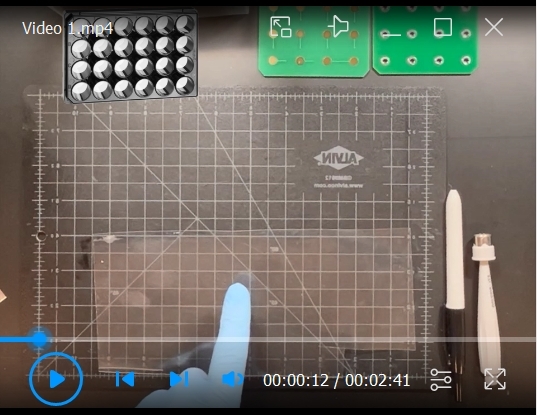
Well-plate electrodes assembly
- **Device assembly**
  1. Immerse the glass cloning cylinders in acetone and sonicate for 10 min. Rinse the cylinders with ethanol and deionized distilled water (DDW) and dry with N_2_ gas.
  2. Use a 6 mm biopsy punch to cut holes (~10 holes for a 75 mm × 25 mm area) through the double-sided adhesive tape (Figure 2B).
  3. Remove one side of the protective film from the adhesive tape using a razor blade to separate the laminate from the tape at a corner and carefully remove the laminate with tweezers (Figure 2C).
  4. Place the track-etched PC membrane on a clean glass slide and place it in oxygen plasma cleaner for 5 min.
  5. Gently place the PC membrane on top of the tape from step B3 ensuring it remains flat by using the rigid microscope slide as support (Figure 2D and E).
  6. Flip the PC membrane–adhesive tape and remove the remaining protective film from the adhesive tape using a razor blade and a tweezer (Figure 2F).
  7. Place the cloning cylinder on the exposed adhesive tape aligned at the location of the pre-cut holes (Figure 2G and H).
  8. Press down on the top of the glass cylinder to activate the pressure adhesive tape and ensure a good seal between the assembled layers (Figure 2I). Repeat this step for each hole cut in the adhesive tape.
  9. Use scissors to cut the tape to separate each cylinder (Figure 2J) and subsequently trim the edge of the tape that surrounds each cylinder (Figure 2K and L). Spray the scissors with 70% ethanol in DI water to prevent the tape from sticking to the surface of the scissors.
  10. Place the devices in a well-plate dish exposed to UV light for 4 h.
  11. LEPD device fabrication and assembly steps are presented in Video 2.
    **Figure 2.**
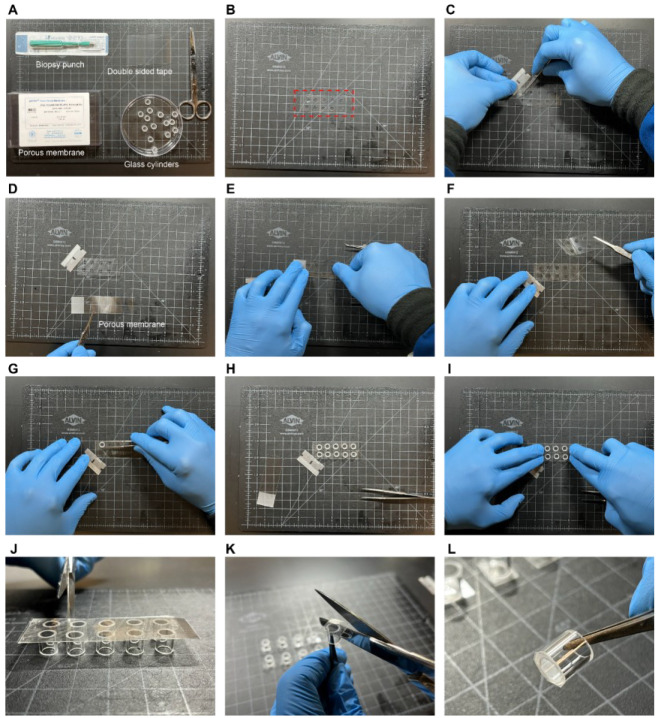
Fabrication of the localized electroporation device (LEPD). (A) Materials needed for the process. (B) Double-sided tape with 6 mm holes punched through. (C) Peeling one side of the adhesive tape. (D) Placing porous membrane on a glass slide. (E) Pressing porous membrane onto the sticky side of the tape. (F) Peeling off the other side of the tape to expose the other side. (G–I) Placing glass cylinders onto the exposed sticky side of the tape and pressing them down to firmly stick. (J) Cutting out each LEPD. (K) Trimming out the edges of LEPDs. (L) Final result of an individual LEPD after completing the process.
    **Video 2.**
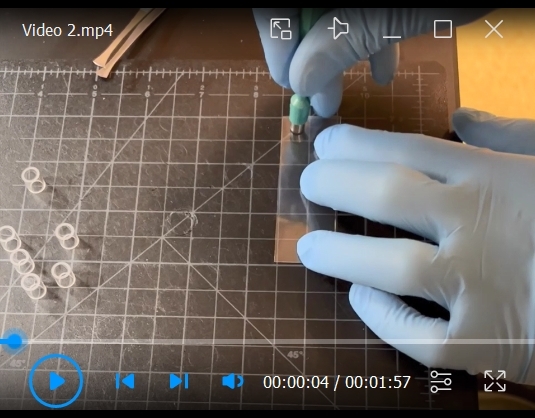
Fabrication and assembly of localized electroporation device (LEPD)
- **Surface treatment and cell culture**
  1. Prepare fibronectin solution for surface treatment by diluting the stock solution in PBS (20 µL of fibronectin in 1 mL of PBS) to obtain a final concentration of 20 µg/mL.
  2. Add 100 µL of the prepared solution to each LEPD and incubate for 1 h at room temperature inside a biosafety cabinet.
  3. Carefully discard the excess, unbound fibronectin solution from each LEPD and wash them with 100 µL of PBS two times. This ensures that only the adhered fibronectin layer is retained on the devices, which are now ready for cell culture.
  4. Prepare complete cell culture media by adding 50 mL (10%) of FBS and 1 mL (1%) of Pen-Strep to 449 mL of DMEM.
  5. Use this media for HeLa cell culture in 6-well plates.
  6. Seed cells at a density of 3 × 10^5^ cells/well and use 2 mL of media per well for culturing.
  7. Wait until cells reach confluency (1 × 10^6^ cells/well) in the well plates before dissociating them for plating in the LEPDs.
  8. When cells are well adhered and confluent, gently discard the cell culture media in the 6-well plates and add 1 mL of warm (37 °C) trypsin-EDTA to each well.
  9. Incubate (at 37 °C with 5% CO_2_) for 5 min.
  10. Gently pipette the trypsin-EDTA to detach and dissociate the cells and transfer the cell suspension to a 15 mL Falcon tube.
  11. Add 4 mL of the complete cell culture media to the cell suspension to neutralize the trypsin.
  12. Centrifuge the cells at 300× *g* for 5 min.
  13. Discard the supernatant, add 1 mL of fresh complete cell culture media, and resuspend the cells.
  14. Count the cells using a hemocytometer and, if necessary, dilute the cells to obtain a final cell suspension concentration of 200 cells/µL.
  15. Add 20,000 HeLa cells in each LEPD by pipetting 100 μL of the cell suspension solution.
  16. Pipette 100 μL of additional complete cell culture media into each well.
  17. Culture the cells on the membrane surface overnight in an incubator (at 37 °C with 5% CO_2_) to promote cell adhesion and tight cell membrane and nanopore contact.
  18. Electroporate the adhered cells on the LEPDs the next day.
  19. Note that this process can be adapted for various adherent and suspension cell types. The surface treatment, cell seeding, and culture conditions must be optimized accordingly.
- **Delivery into suspension cells**
  1. Check the cells in the microscope to ensure they look viable and have proper morphology.
  2. Count the cell density using a hemocytometer or automated cell counter.
  3. Take the appropriate volume of cells from the media to plate between 30,000 and 50,000 cells per device (e.g., 24 devices require ~1.2 million cells).
  4. Take the appropriate volume of media that contains the desired number of cells calculated from the cell density obtained from step D2 and place the cells in a Falcon tube.
  5. Place the tube containing the cells in a centrifuge and add a counterweight to balance. Centrifuge at 150× *g* for 5 min.
  6. Gently remove the cell media from the Falcon tube, leaving the pellet of cells. Add electroporation buffer to the tube: 100 μL of electroporation buffer per device (2.4 mL for 24 devices). Mix the cells in the electroporation buffer.
  7. Dispense 100 μL of electroporation buffer containing the cells in each device inside of a well-plate.
  8. Place the well plate that contains the devices with cells into the centrifuge. Place a counterweight (a well plate with ~200 μL fluid in each well) in the opposite chamber of the centrifuge and centrifuge at 150× *g* for 5 min.
- **Optimization of plasmid delivery**
  1. Prepare eGFP plasmid solutions of concentration ranging from 100 ng/µL to 350 ng/µL with a step size of 50 ng/µL. The minimum volume of each solution should be 5 µL.
  2. Take the electroporation buffer out of 4 °C storage and allow it to reach room temperature.
  3. Transfer the LEPDs with the cells from the incubator into a biosafety cabinet. Carefully pipette out all the media from the LEPDs and add 200 µL of the electroporation buffer to each LEPD.
  4. If using non-adherent cells, place the LEPDs from step E3 in a well plate and centrifuge them at 150× *g* for 5 min.
  5. Using a 10 µL pipette, place a 5 µL droplet of the 100 ng/µL eGFP plasmid solution on the bottom gold electrodes of the wells of column 1 of each row of the 24-well LEPD system. Repeat this for the higher concentration eGFP plasmid solutions for the remaining columns going up in concentration to 350 ng/µL for the sixth column (Video 3).
    **Video 3.**
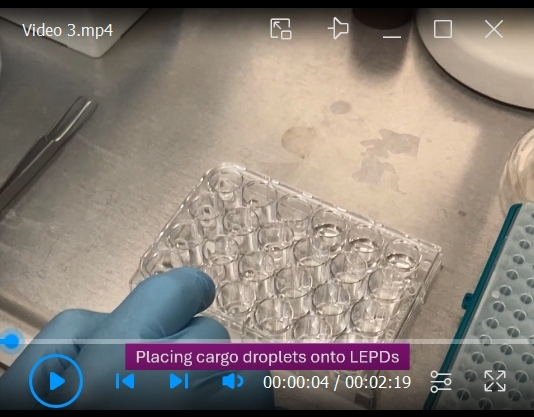
Loading, transmitting, and voltage optimization of cargo delivery
  6. Using a pair of sterile tweezers, gently place the LEPDs with the cultured cells onto the wells with the droplet of delivery plasmid on the bottom gold electrode with one LEPD device per well, as shown in Video 3.
  7. Set the pulse conditions on the electronics software (provided by Infinitesimal LLC) to a bi-level pulse with the following parameters: V1 ranging from 10 to 40 V, V2 fixed at 10 V, T1 at 0.5 ms, T2 ranging from 0.5 to 2.5 ms, frequency set at 20 Hz, and pulse count between 100 and 800. For guidance on using the software, refer to Video 3.
  8. Connect the positive terminal of the electronics box to row 1 of the top plate and the negative terminal to the bottom plate using cables with alligator clip connectors.
  9. Check the resistance of the system using the Infinitesimal LLC software and check for loose connections if the resistance is not in the 1–2 MΩ range and is unstable.
  10. Use the electronics software to apply the pulse; the cells in the devices in row 1 will be subjected to localized electroporation.
  11. After the pulse (~2 min) ends, switch the positive terminal to the next row and repeat steps E7–10. These steps need to be repeated until cells in all four rows are subjected to electroporation.
  12. Disconnect the electrical connections, lift the top plate, and transfer all the LEPDs into a new transparent well plate.
  13. Carefully pipette out the electroporation buffer from all the devices, add fresh cell culture media to the LEPDs, and transfer them into the incubator.
  14. This protocol for optimizing plasmid concentration along the columns and pulse parameters along the rows can be repeated for as many combinations as we want to try.
- **Automated imaging**
  1. To clean the bottom of the LEPD membrane that comes in contact with the delivery reagents, prepare a 12-well plate by dispensing 1 mL of PBS in each well. Dip the LEPDs in the wells without fully submerging the devices (three wells sequentially) and transfer the clean LEPDs to a transparent 24-well plate for imaging.
  2. To quantify cell viability and delivery efficiencies, prepare a solution of Hoechst 33342 nuclear stain in PBS (0.1 mg/mL), gently aspirate the fluid from each well (cell media or electroporation buffer) without completely drying the well, and place 100 µL of Hoechst solution prepared in the well for 10 min.
  3. Gently aspirate the Hoechst solution, wash thrice with PBS, and dispense 150 µL of PBS for imaging.
  4. Place the well plate containing the LEPDs in a motorized microscope stage.
  5. Program the stage to move to the center of each well in the plate and use an objective lens with 10× or 20× magnification to capture images of the stained nuclei using a DAPI filter.
  6. Focus on the cells manually using the coarse and fine focus knobs or by programming the microscope with an autofocus routine. Briefly, the autofocus routine consists of calculating the focus score (e.g., normalized variance, Laplacian, or log-histogram) for a Z-stack of images acquired in a single field-of-view, moving to the Z-plane with the highest score, and iteratively reducing the step size and scanning range until achieving optimal focus. To reduce the time of the routine, the user manually focuses on the first well to set a reference Z-plane to be used for the subsequent wells, since the difference in focus is small between wells.
  7. Obtain images with multiple fluorescence and brightfield filters for each field of view. The choice of filters depends on the excitation and emission characteristics of the fluorescent probe to be examined.
  8. For each LEPD, acquire images at multiple fields of view by moving manually or by programming the microscope to move to different locations within each well.
  9. The representative transfection images of adherent and suspended cell lines, HeLa and K562, after plasmid (pmax GFP) delivery are presented in Figure 3A–B. The transfection efficiency appears to be lower in K562 cells, likely due to their non-adherent nature, which hampers efficient contact with the porous cell membrane. Figure 4A–B provides the viabilities images taken 24 h post-transfection for HeLa and HEK 293T cell lines. Further discussion of these images can be found in Patino et al. [9].
    **Figure 3.**
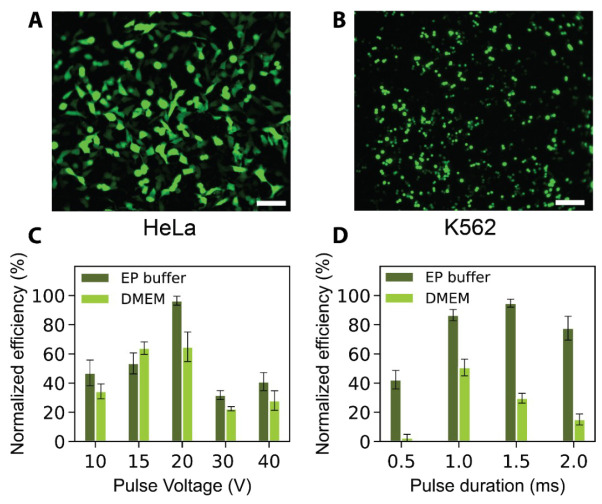
Representative efficiency results. Representative images of successful transfection of fluorescent protein-encoding plasmids into (A) HeLa (adherent) and (B) K562 (cells in suspension) transfected with a pmax GFP–encoding plasmid. Scale bars represent 100 µm. Variation of transfection efficiency for HEK 293T cells electroporated in cell culture media (DMEM) and EP buffer using a localized electroporation device (LEPD) with respect to (C) pulse voltage and (D) pulse duration. All error bars indicate the standard error of the mean (SEM) of triplicate samples, n _cell_ > 100 per sample for all bar plots. All transfection efficiencies are normalized with respect to the highest value of efficiency in each plot. The highest efficiencies for plots in A and B are 71.6% and 63.6%, respectively. Figures are adapted from Patino et al. [9].

## Data analysis

**Image segmentation and data analysis**

1. To identify the cells in the images, a fully convolutional network (FCN) [25] with a U-Net architecture [26] was trained using images and corresponding labels of various cell types and imaging modalities (e.g., fluorescence and phase contrast). The FCN network consists of an encoder-decoder architecture containing 20 hidden layers (e.g., convolution, pooling, and up-convolution) that enables the classification of objects in the images (e.g., cell exterior, nucleus) with pixel-level resolution [20,27].
2. To train the U-Net for a specific cell type of interest, images of cells were manually labeled using drawing tools in image manipulation software. To facilitate labeling of the cell exterior or internal cell compartments of interest, the cells were stained with fluorescent dyes (e.g., Hoechst nuclear dye or Calcein AM cytosolic dye) and imaged as described in section F.
3. The images and their corresponding ground truth labels were split into training, validation, and test sets to cross-validate the training process and prevent overfitting.
4. A weighted soft-max cross-entropy loss function was used to classify each pixel into three categories (interior, exterior, and boundary).
5. The U-Net was optimized using stochastic gradient descent (momentum = 0.9, learning rate = 1 × 10^-4^). The training was performed using a graphic processing unit (GPU: NVIDIA) to expedite the process for a large network containing more than 7.5 million trainable parameters.
6. To assess the performance of the model, the area overlap between the predicted objects and the ground truth labels was measured and used to determine true positives (TP), false positives (FP), true negatives (TN), and false negatives (FN). From these values, the precision, recall, and F1 scores could be calculated as follows:
precision = TP/(TP+FP), recall = TP/(TP+FN), F1 = 2 × (precision × recall)/(precision + recall)
7. For analysis of viability, delivery, and transfection efficiencies, the Hoechst nuclear images were used to segment the nuclei of the cells to determine the number of cells in each image. Moreover, the segmented nuclei were used as a mask to measure the fluorescence intensity of the other color channels (e.g., GFP filter for eGFP transfection and TexasRed filter for quantification of dead cells using propidium iodide). The fluorescence intensity for the respective filters was compared to negative control samples to determine the threshold for a positive signal. Viability and transfection efficiency were calculated as follows:
$$viability={1-(N}_{dead}/N_{cells})$$
$$efficiency=N_{transfected}/N_{cells}$$
8. To extract measurements from the segmented images, a cell analysis software (CellProfiler) was used to obtain numerous features (e.g., shape, intensity, texture) from each cell.
9. Figure 3C and D illustrate the efficiencies of GFP plasmid delivery into HEK 293T cells under varying voltages and pulse durations, and across different buffer conditions. The post-transfection viability results of HEK 293T cells with different voltages are shown in Figure 4C, where viability remains high between 10 and 20 V and declines at higher voltages of 30 and 40 V in both EP and DMEM buffer. Figure 4D represents the viability results for HeLa, HEK, and K562 cell lines post-transfection. Additional data and further discussions are available in Patino et al. [9].
10. The features were transformed using the generalized logarithm (glog) method and standardized using the median and median absolute deviation (MAD) to obtain the robust Z score (R.Z. score). In contrast to the Z-score, the R.Z. score is not sensitive to outliers.
11. To analyze the features, correlation matrices and 2D feature projection maps (U-Map, t-SNE) can be applied.
  **Figure 4.**
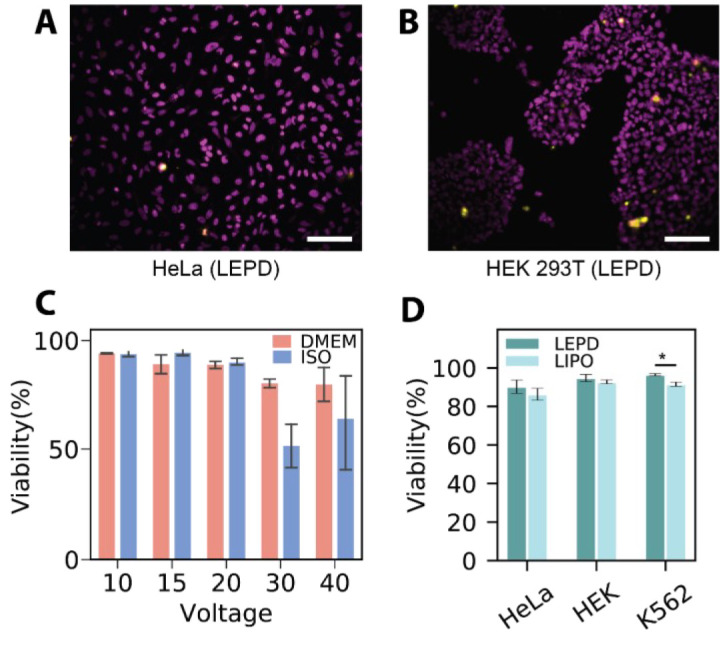
Representative viability results. Representative composite fluorescence micrographs of propidium iodide (PI) viability assay conducted on (A) HeLa and (B) HEK 293T 24 h after being treated with the localized electroporation device (LEPD); pseudo colors: magenta—Hoechst, yellow—PI. Scale bar = 100 µm. (C) Viability after plasmid delivery across different voltages: Bar plots show the mean viability of HEK 293T cells transfected using the LEPD in both DMEM and EP buffer in the voltage range 10–40 V. (D) Viability of various continuous cell lines 24 h after treatment with LEPD and LIPO. All error bars indicate the standard error of the mean of triplicate samples, n_cell_ > 100 per sample for all bar plots. Figures are adapted from Patino et al. [9].

## Validation of protocol

This protocol or parts of it has been used and validated in the following research article(s):

- Patino et al. [9]. Multiplexed high-throughput localized electroporation workflow with deep learning–based analysis for cell engineering. *Sci Adv.* (Figures 4–7).
